# Comparing the effects of a 7-week repeated sprint training in hypoxia vs. heat in males and females

**DOI:** 10.3389/fspor.2026.1833950

**Published:** 2026-05-21

**Authors:** Anna Piperi, Geoffrey Warnier, Laura Schmit, Marc Francaux, Louise Deldicque

**Affiliations:** Institute of Neuroscience, UCLouvain, Louvain-la-Neuve, Belgium

**Keywords:** maximal oxygen consumption, power output, repeated sprint ability, sex, Wingate test

## Abstract

**Introduction:**

This comparative analysis aimed to determine whether repeated sprint training in hypoxia (RSH) or heat (RSHT) more effectively improves repeated sprint ability (RSA) in normoxic, temperate conditions, while also examining potential sex differences.

**Methods:**

Males and females participated in two randomized controlled studies consisting of a 7-week repeated sprint training 2×/week in hypoxia (HYP; F_i_O_2_ = 0.146, 18–20°C, 55% relative humidity (RH); males: *n* *=* 12, females: *n* *=* 10), heat (HEAT; F_i_O_2_ = 0.209, 30°C, 60% RH; males: *n* *=* 12, females: *n* *=* 14), or control conditions (CON; F_i_O_2_ = 0.209, 18–20°C, 55% RH, males: *n* *=* 23, females: *n* *=* 21). RSA and aerobic and anaerobic capacities were evaluated in normoxic, temperate conditions before and after training.

**Results:**

The delta (*Δ*) from Pre- to Post-test was compared between training environmental conditions and sexes for the variables showing improvements after training. The *Δ* in sprint number during the RSA test was higher in the intervention than the control group (HYP vs. CON, *Δ* =  + 6 ± 1 sprints, *p* < 0.001; HEAT vs. CON, *Δ* =  + 4 ± 1 sprints, *p* = 0.001) with no difference between HYP and HEAT. A tendency for a main sex effect (*p* = 0.071), and for a sex*ENV interaction (*p* = 0.070) was observed. The *Δ* in power output during the RSA test, mean power during the Wingate test, and $\dot{V}O_{2}$ peak showed no differences between environmental conditions or sexes, while a tendency for a main sex effect in RSA total work was detected (*p* = 0.055).

**Discussion:**

Seven weeks of RSH and RSHT similarly enhanced RSA in males and females under normoxic, temperate conditions, with a trend towards further improvements with hypoxia in males.

## 1 Introduction

Over the past decades, the participation of women in sports has grown substantially across all levels (1). Despite this rise, women remain underrepresented in exercise physiology research, limiting the development of evidence-based, sex-specific training guidelines (2). Given the physiological and hormonal differences between men and women, it is essential to investigate whether training strategies are equally effective across sexes (3). For many sports, including team, racket, and combat sports, enhancing repeated sprint ability (RSA) is of major importance, as it could influence the outcome of a game (4, 5). Indeed, these intermittent disciplines are characterized by repeated short-duration sprints (<10 s), interspersed with brief recovery periods (<30 s) (6). Therefore, coaches are seeking time-efficient strategies to improve the athlete's capacity to recover quickly and sustain performance across multiple sprints.

Recently, combining repeated sprint training with environmental stressors, such as hypoxia or heat, has gained popularity as a promising approach to improve RSA in sea-level and temperate conditions (7, 8). On the one hand, repeated sprint training in hypoxia (RSH) has been intensively investigated over the past 12 years and has been found to enhance RSA through vascular and molecular adaptations (9). Those adaptations include increased blood perfusion which may support better waste removal and delayed fatigue (9). At the molecular level, RSH was shown to improve anaerobic glycolytic capacity, with upregulation of glycolytic and pH-regulating gene expression and downregulation of the expression of mitochondrial biogenesis markers (9). On the other hand, repeated sprint training in heat (RSHT) and its effects on RSA were less explored in the literature (7). Some studies suggest that RSHT might induce partial heat acclimation and therefore improve intermittent sports performance via improvements in core temperature (10), thermal sensation (10), sweat electrolyte concentration (11), and heart rate (11). Both training interventions seem to enhance performance; however, it remains unclear which type of training is more effective for improving RSA.

Interestingly, a review by Baranauskas et al. (2021) compared the effects of hypoxic vs. heat training on endurance performance at sea level and thermoneutral environment (12). The authors suggested that even though both modalities offer distinct adaptations, hypoxic training provides clearest benefits and it is therefore the preferred environmental stimulus for endurance athletes (12). However, this review mainly examined protocols that consist of longer and daily exposures to hypoxia or heat (such as altitude or heat camps), while typical sessions designed to improve RSA involve shorter and intermittent exposures. Moreover, it focused on endurance training and performance, not repeated sprints, and did not address potential sex-specific differences. Evidence suggests that females might respond differently than males to RSH due to sex differences in fatigue resistance (13) and exercise-induced hypoxemia (14). More specifically, it has been reported that males experienced a greater performance decrement than females during repeated sprints, likely due to the greater initial power output and greater reliance of anaerobic glycolysis in males (13). In addition, it has been observed that exercise-induced hypoxemia was greater in females than males when exposed to hypoxic conditions, most likely due to their lower hemoglobin concentrations and smaller lung volumes (14). Regarding heat acclimation, the overall physiological adaptations appear similar between males and females, but their magnitude and time course might be affected by the sex differences in thermoregulation (15). For example, it has been reported that the rise in circulating progesterone during the luteal phase increases resting body temperature by approximately 0.3–0.5 °C (16). As a result, less metabolic heat needs to be generated to reach the adaptation threshold of 38.5 °C (17). This may reduce the overall thermal stress experienced during training, potentially limiting the extent of physiological adaptations and subsequent performance gains (15). Moreover, sex-related differences may exist in the time course of heat acclimation. Research indicates that, compared to males, females may need either longer daily heat exposures or a greater number of sessions to achieve similar reductions in cardiovascular and thermoregulatory strain (18). Given the dependency of adaptations on the magnitude and duration of exposure (19, 20), the differences between endurance and sprint performance, such as the greater reliance on neuromuscular and anaerobic components in RSA (21), and the potential sex differences in training adaptations (3), the findings from the review of Baranauskas et al. (2021) cannot be extrapolated to sprint-based training.

To date, no research in either males or females has directly compared the effectiveness of hypoxic vs. heat stressors combined with repeated sprint training on improving repeated sprint ability in normoxic and thermoneutral conditions. Therefore, the present study aims to directly compare RSH vs. RSHT to determine which strategy is more effective in improving repeated sprint ability under normoxic and temperate conditions, while also exploring potential sex-based differences in performance responses.

## 2 Materials and methods

### 2.1 Comparative analysis overview

This is a comparative analysis of two previously randomized controlled studies conducted by our research group, one published (22) and the other submitted for publication (23). Both studies examined the effects of repeated sprint training in combination with an environmental stressor, either hypoxia (HYPOXIA study) (22) or heat (HEAT study (23), unpublished manuscript), on RSA in active males and females. While the studies were conducted independently and on different individuals, the screening process, the training and testing protocols, as well as participant characteristics were closely matched, allowing for a *post-hoc* comparison. Moreover, both studies were approved by the same institutional ethics committee and conducted in accordance with the Declaration of Helsinki. Details regarding the design, and training and testing protocols of the two studies are shown in the sections below.

#### 2.1.1 Design of the HYPOXIA study and the HEAT study

**Figure 1:**
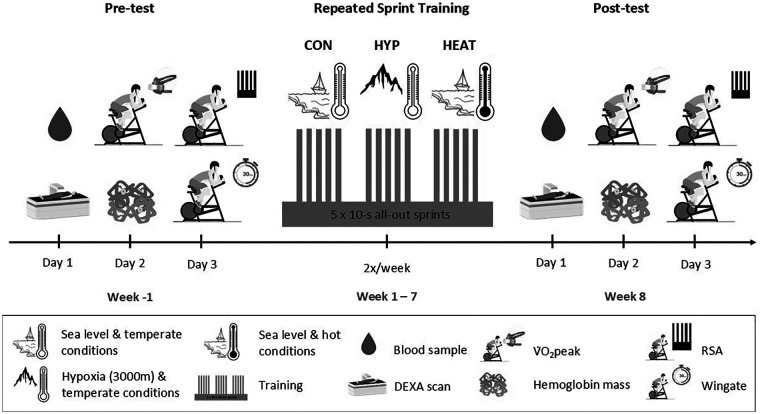
Design of the two studies included in the comparative analysis.

The HYPOXIA study was conducted between April 2022 and June 2023, while the HEAT study was conducted between February 2024 and December 2024, excluding the summer months July and August. Outside mean monthly temperature was 10.8 ± 3.7°C. Each study consisted of three phases: one week of Pre-test (week -1), 7 weeks of supervised training (weeks 1–7), and one week of Post-test (week 8) (Figure 1). Training involved 14 visits whereas each of the Pre- and Post-test phases involved 3 visits to the laboratory, resulting in a total of 20 visits per participant. During test phases, the following tests and measurements were performed in normoxic and temperate conditions, to provide a comprehensive assessment of several physiological variables and support performance data. Visits 1 and 18 (day 1 of experimental trial): body mass, height, body composition assessment and blood sampling; Visits 2 and 19 (day 2): $\dot{V}O_{2}$ peak and hemoglobin mass (Hb mass) measurements; Visits 3 and 20 (day 3): RSA test, muscle oxygenation measurement and Wingate test. During visit 1, participants were assessed for eligibility through medical screening and questionnaires, after providing written consent. If eligible, participants were then randomly assigned to one of the two intervention groups in each study, with repeated sprint training in either normoxic/control (NOR) or hypoxic (HYP) conditions in the HYPOXIA study and temperate/control (CON) or hot (HEAT) conditions in the HEAT study.

#### 2.1.2 Participants

A sample size analysis was performed to determine the optimal number of participants. To find an 8% increase in the mean power output during the RSA test with a 12% SD and a dropout rate of 10%, it was predicted that 48 participants (*n* = 12/group) were needed. A total of 92 healthy, recreationally active males and females (age 18–40 years, engaging in physical activity minimum 3 h/week for at least 6 months prior to the experiments) completed all experimental procedures. The HYPOXIA study included 41 participants (Males-NOR, *n* = 11; Females-NOR, *n* = 8; Males-HYP, *n* = 12; Females-HYP, *n* = 10), while the HEAT study included 51 participants (Males-CON, *n* = 12; Females-CON, *n* = 13; Males-HEAT, *n* = 12; Females-HEAT, *n* = 14). For this comparative analysis, the control groups of the HYPOXIA study (NOR) and the HEAT study (CON) are presented merged in Table 1 (CON), to compare baseline characteristics between environmental conditions (ENV: control, hypoxic and hot conditions) and sexes (males and females). On each test day, female participants were asked to carry out an ovulation test (Sensitest LH, Sensitest, EJ Delfgauw, The Netherlands), as just before ovulation, 17 beta estradiol levels increase and can have a positive effect on performance (24). In case of a positive ovulation test, testing would have been rescheduled, however all participants presented a negative ovulation test. Thirteen out of eighteen females in the HYPOXIA study and fourteen out of twenty-seven in the HEAT study were using hormonal contraception, which represents 60% of the total number of females. Additionally, concentrations of 17 beta estradiol in females were determined by ELISA. In both studies, concentrations of 17 beta estradiol did not differ between Pre- and Post-test (HYPOXIA: 26.7 ± 6.2 vs. 31.3 ± 12.3 pg·mL^−1^, *p* = 0.500 and HEAT: 43.4 ± 8.7 vs. 39.1 ± 8.3 pg·mL^−1^, *p* = 0.394).

**Table 1:** Participants characteristics.

	CON		HYP		HEAT	
	Males (*n* = 23)	Females (*n* = 21)	Males (*n* = 12)	Females (*n* = 10)	Males (*n* = 12)	Females (*n* = 14)
Age (y)	24.2 ± 0.9	24.5 ± 1.2	24.4 ± 1.2	24.4 ± 0.9	25.7 ± 1.3	25.8 ± 0.7
Body weight (kg)	74.1 ± 2.6	61.1 ± 2.0***	72.9 ± 1.6	66.9 ± 1.6**	77.4 ± 3.9	61.1 ± 1.7**
Height (cm)	180 ± 2	166 ± 2***	179 ± 2	167 ± 2***	182 ± 1	169 ± 2***
Training volume (h·wk^−1^)	8.2 ± 0.8	5.6 ± 0.5*	9.5 ± 1.2	8.7 ± 1.3	8.6 ± 0.9	6.2 ± 0.8
LBM (kg)	63.9 ± 1.8	45.3 ± 1.4***	62.2 ± 1.3	48.6 ± 1.1***	67.3 ± 1.9	46.9 ± 1.3***
Fat percentage (%)	11.3 ± 0.8	24.0 ± 1.0***	12.5 ± 1.2	25.7 ± 1.4***	10.3 ± 1.7	21.3 ± 1.4***
Basal $\dot{V}O_{2}$ peak (mL·kg^−1^·min^−1^)	49.7 ± 1.3	40.3 ± 1.3***	50.1 ± 1.8	39.2 ± 1.9***	55 ± 3	41 ± 2***
Basal $\dot{V}O_{2}$ peak (mL·kg^−1^ LBM·min^−1^)	57.3 ± 1.4	54.1 ± 1.6	59.3 ± 1.9	54.0 ± 2.3	62 ± 2	53 ± 2*
Basal PP 10-s sprint (W)	852 ± 36	615 ± 27***	874 ± 36	676 ± 32***	937 ± 50	673 ± 38**
Basal PP 10-s sprint (W·kg^−1^ LBM)	13.3 ± 0.3	13.6 ± 0.4	14.1 ± 0.5	13.9 ± 0.6	13.9 ± 0.5	14.3 ± 0.5

CON, normoxic (F_i_O_2_ 0.209) and temperate (20 °C, 55% relative humidity (RH)) conditions; HYP, hypoxic (F_i_O_2_ 0.146) and temperate conditions; HEAT, hot (30 °C, 60% RH) and normoxic conditions; LBM, lean body mass; PP, peak power output. Values are means ± SEM.

* *p* < 0.05.

** *p* < 0.01.

*** *p* < 0.001 for difference with males in same environmental conditions.

Inclusion and exclusion criteria were consistent across studies. Participants in the HYPOXIA study were performing intermittent sports (e.g., football, volleyball, boxing, tennis, etc.) while participants in the HEAT study were practicing various sports disciplines, such as intermittent sports (e.g., football, volleyball, boxing, tennis, etc.) and/or endurance sports (e.g., running, cycling, swimming, etc.). Exclusion criteria for participation were smoking, exposure to an altitude ≥1,500 m during the month before the HYPOXIA study or exposure to a temperature above 25°C for more than 7 consecutive days during the month prior to the beginning of the HEAT study, any health conditions or injuries that could compromise the participant's safety during training/testing, prescribed medication, and performing additional repeated sprint training outside their training sessions and matches of their sport discipline more than once per week.

#### 2.1.3 Training interventions

Participants in both studies completed a repeated sprint training protocol on a cycle ergometer (Cyclus II, RBM Electronics, Leipzig, Germany) twice a week for 7 weeks, as described by Faiss et al. (9). Three to four days of rest were allowed between the training sessions and the test phases. Training sessions took place in an environmental chamber built in our laboratory (High altitude system B-Cat, Tiel, The Netherlands). The control group in both studies (NOR and CON) completed the training sessions in normoxic (F_i_O_2_: 0.209) and temperate conditions (18–20 °C, 55% relative humidity (RH)). The only difference between studies was the environmental condition during the training sessions of the intervention group. The intervention group in the HYPOXIA study trained in hypoxic (F_i_O_2_: 0.146 to simulate an altitude of 3,000 m by injecting nitrogen in the chamber) and temperate conditions (18–20 °C, 55% RH). The intervention group in the HEAT study trained in heat (30 °C, 60% RH) and normoxic conditions (F_i_O_2_: 0.209).

The training sessions started with a 10-min warm up at 50% of $\dot{V}O_{2}$ peak including two 5-s sprints (torque = 4.2 N·kg^−1^ of lean body mass (LBM)) at min 5 and 8, followed by 3 sets of 5 × 10-s all-out repeated sprints and ending with a 10-min cool down on the cycle ergometer. An active recovery was allowed for 20 s between sprints and for 5 min between sets. The torque used during the sprints was set at 4.2 N·kg^−1^ of LBM. Of note, based on preliminary results from our laboratory and on two previous articles, one in males (9) and another one in females (25), we defined 6.5% of the subject's body mass as optimal training intensity, which corresponds to an intensity of 4.2 N·kg^−1^ of LBM in our subjects. Participants were instructed to go “all-out” during each sprint and try to produce the highest power output possible. During the active recovery and cool down, resistance was set at 30% of $\dot{V}O_{2}$ peak. At week 7, the number of sets decreased to two and sprint torque was reduced to 3.7 N·kg^−1^ of LBM to favor recovery before the Post-test phase. Thermoregulation measures were taken during training 1 and 12 of the HEAT study only. Water was provided *ad libitum* to all participants to ensure appropriate hydration during training. Each session lasted about 40 min (30 min on week 7) in the chamber (540 min in total during the 7-wk period). In the HEAT study only, prior to the start of the warm-up, participants were seated passively in the chamber for 20 min, resulting in a 60-min session (820 min in total). The total sprint time for both studies was 33.3 min. At least one day of rest was allowed between the two weekly sessions, to ensure optimal recovery. Participants were asked to maintain their usual training volume outside the laboratory throughout the duration of the study.

#### 2.1.4 Pre- and post-tests

Participants in both studies were asked to follow a similar diet during the three days preceding each test at Pre- and Post-test phases. They were also asked to abstain from alcohol and caffeine during the 24 h before each test and from intense physical exercise during the 48 h before. They were advised to consume a standard meal prior to the tests and reproduce the same meal for Pre- and Post-test phases. They were also asked not to modify their eating habits during the study period.

A cycle ergometer (Cyclus II, RBM Electronics, Leipzig, Germany) was used for all exercise performance tests i.e., VO_2_peak, RSA and Wingate tests. The seat position was adjusted for each participant and reproduced for all tests. Pre- and Post-test was performed in the exact same order in normoxic (F_i_O_2_, 0.209) and temperate (18–20 ^°^C, 55% RH) conditions in both studies.

Several tests and measurements were performed Pre- and Post- training using identical protocols in both studies. Since this comparative study aims to determine which environmental training method is more effective in males and females, the following methods section and analysis focus solely on the variables that demonstrated a training effect (improvement) in the HYPOXIA study (22) and/or the HEAT study (23). Those variables include the number of sprints (HYPOXIA: *p* < 0.001, HEAT: *p* = 0.001), total work (HYPOXIA: *p* < 0.001; HEAT: *p* = 0.002) and average and peak power output during the RSA test (HEAT: *p* = 0.003, *p* = 0.021, respectively), average power during the Wingate test (HYPOXIA: *p* = 0.036; HEAT: *p* = 0.007) and $\dot{V}O_{2}$ peak (HYPOXIA: *p* = 0.039; HEAT: *p* = 0.032) (Table 2).

**Table 2:** Tests performed in normoxic and temperate conditions that demonstrated a training effect in the HYPOXIA and/or the HEAT study following repeated sprint training.

Hypoxia study									
	Males – NOR (*n* = 11)		Females – NOR (*n* = 8)		Males – HYP (*n* = 12)		Females – HYP (*n* = 10)		Training effect
	Pre	Post	Pre	Post	Pre	Post	Pre	Post	*p*-value
RSA sprint no	11 ± 1	13 ± 2	8 ± 1	10 ± 1	11 ± 1	21 ± 2*	8 ± 1	14 ± 2*	**<0**.**001**
RSA TW (kJ)	58 ± 4	64 ± 3	29 ± 3	36 ± 2	56 ± 3	71 ± 2	32 ± 3	41 ± 1	**<0**.**001**
RSA MP (W)	632 ± 22	658 ± 23	431 ± 26	457 ± 26	623 ± 20	653 ± 20	489 ± 19	507 ± 16	0.108
RSA PP (W)	869 ± 35	864 ± 43	611 ± 30	615 ± 32	874 ± 36	878 ± 44	676 ± 32	694 ± 23	0.835
Wingate MP (W·kg^−1^)	8.6 ± 0.2	9.0 ± 0.3	6.6 ± 0.3	7.1 ± 0.2	9.0 ± 0.2	9.2 ± 0.2	7.1 ± 0.3	7.4 ± 0.3	**0**.**036**
$\dot{V}O_{2}$ peak (mL·kg^−1^·min^−1^)	49 ± 2	52 ± 2	38 ± 2	40 ± 2	50 ± 2	54 ± 2	39 ± 2	43 ± 2	0.039

Heat study									
	Males – CON (*n* = 12)		Females – CON (*n* = 13)		Males – HEAT (*n* = 12)		Females – HEAT (*n* = 14)		Training effect
	Pre	Post	Pre	Post	Pre	Post	Pre	Post	*p*-value
RSA sprint no	14 ± 2	16 ± 2	9 ± 1	11 ± 1	15 ± 1	21 ± 3*	8 ± 1	13 ± 2*	**0**.**001**
RSA TW (kJ)	71 ± 5	80 ± 5	33 ± 2	41 ± 3	85 ± 5	95 ± 4	33 ± 2	38 ± 2	**0**.**002**
RSA MP (W)	570 ± 25	642 ± 30	456 ± 20	490 ± 20	661 ± 18	689 ± 17	473 ± 17	515 ± 16	**0**.**003**
RSA PP (W)	869 ± 59	956 ± 55	661 ± 37	743 ± 46	984 ± 51	1,028 ± 46	700 ± 34	779 ± 25	**0**.**021**
Wingate MP (W·kg^−1^)	8.9 ± 0.2	9.6 ± 0.1	7.4 ± 0.1	7.8 ± 0.1	9.2 ± 0.3	9.4 ± 0.3	7.7 ± 0.2	8.0 ± 0.2	**0**.**007**
$\dot{V}O_{2}$ peak (mL·kg^−1^·min^−1^)	50 ± 2	52 ± 2	42 ± 2	44 ± 2	55 ± 3	57 ± 3	41 ± 2	45 ± 2	**0**.**032**

CON, temperate conditions (18–20 °C, 55% relative humidity (RH)); HEAT, hot conditions (30 °C, 60% RH); MP, mean power output; PP, peak power output; VO_2_peak, peak in oxygen consumption measured during incremental exercise test. Values are means ± SEM.

* *p* < 0.05 for difference with control group within each study; *p*-values in bold denote statistical significance.

#### 2.1.5 $\dot{V}O_{2}$ peak test

On the second day of the tests, aerobic capacity was assessed using an incremental exercise test. The starting load was set at 1.2 W·kg^−1^ of LBM and was incremented by 0.6 W kg^−1^ of LBM every 2 min until exhaustion. Respiratory exchanges were monitored throughout the test (Ergocard Clinical, Medisoft, Sorinnes, Belgium). Peak oxygen uptake ($\dot{V}O_{2}$ peak) was defined as the highest average value over 30 s during the test. Participants were instructed to maintain a cadence of 75–85 rpm while strong verbal encouragement was given throughout the test.

#### 2.1.6 Repeated sprint ability test

On the third day of the tests, participants performed a 10-min warm-up at 50% of $\dot{V}O_{2}$ peak, including two 5-s all-out sprints at min 5 and 8 (torque = 0.8 Nm·kg^−1^ of body mass). Participants then performed the RSA test, which consisted of 10-s all-out sprints against a fixed torque of 0.8 Nm·kg^−1^ of body mass with a 20-s active recovery between sprints at 30% of $\dot{V}O_{2}$ peak. Participants were instructed to go “all-out” during each sprint and perform as many sprints as possible until exhaustion. Strong verbal encouragement was given throughout the test but no indication of the number of sprints performed. Volitional exhaustion or minimal pedaling frequency of 70 rpm for more than 5 s during a sprint were the criteria to stop the test, which was then followed by a 10-min cool down at 30% of $\dot{V}O_{2}$ peak. Mean and peak power output was registered and total work (TW), which reflects the mechanical work spent during the sprints, was calculated as:

$$\mathrm{TW}(\mathrm{kJ})=\left[\frac{\left(S_{1\;}\times \;10)+(S_{2\;}\;\times \;10)+(S_{3}\;\times \;10)+\;\ldots +(S_{\mathrm{final}}\times \;10\right)}{1000}\right]\;$$

Of note, total work and power output were calculated on the same number of sprints for Pre- and Post-tests, i.e., the number of sprints performed at Pre-test, to reduce the large interindividual differences induced by the nature of the RSA test, i.e., unfixed number of sprints to exhaustion.

#### 2.1.7 Wingate test

One hour after the RSA test, participants performed a warm-up as described previously. Then, they completed a standard 30-s Wingate test with a resistance set to 7.5% of the participant's body mass (0.075 kg·kg^−1^ of body mass). Participants were asked to generate the highest power output possible throughout the entire test, and they received strong verbal encouragement. Residual fatigue from the RSA test was likely present. However this test was performed for all participants at the same moment of the testing sequence, as done previously by Faiss et al. (2013) (9), allowing for a good comparison of the participants' ability to perform a 30-s all out sprint. Moreover, the Wingate test was performed at the same timing with respect to the RSA test at Pre- and Post-test.

### 2.2 Statistics

Baseline characteristics data of the subjects were assessed for normality using the Shapiro–Wilk test. Between groups comparisons were performed using one-way ANOVA or the non-parametric Kruskal–Wallis test accordingly. Linear mixed models were applied on the delta between the Pre- and Post-tests data of variables measured during the RSA, Wingate and $\dot{V}O_{2}$ peak tests in the HYPOXIA and the HEAT studies. Sex and environmental condition were used as fixed factors. The main effects of sex and environmental condition, and the interaction sex*ENV (differences between males and females in CON and/or HEAT) were reported. In case of significant ENV effect, the Sidak *post-hoc* test was applied for pairwise multiple comparisons. The *post-hoc* analyses were presented with a 95% confidence interval (CI = lower limit, upper limit). Linear mixed models give unbiased results in the presence of missing data and take potential differences at baseline into account. Effect size was determined for main effects using the partial eta squared (*η_p_*^2^), and was classified as small (≤0.01), medium (0.02–0.06), large (0.07–0.14) and very large (>0.14) (26). Where the main effect was not significant, only the *p*-value and effect size were reported. Significance level was set at *p* < 0.05. All statistical analyses were performed using the Statistical Package for the Social Sciences (SPSS v.28.0.1.1, IMB, Armonk, NY, USA). All graphs were generated using the GraphPad Prism (v.9.0.0, GraphPad Software, Boston, USA). Values are expressed as means ± SEM. Individual values are shown on graphs.

## 3 Results

**Figure 2:**
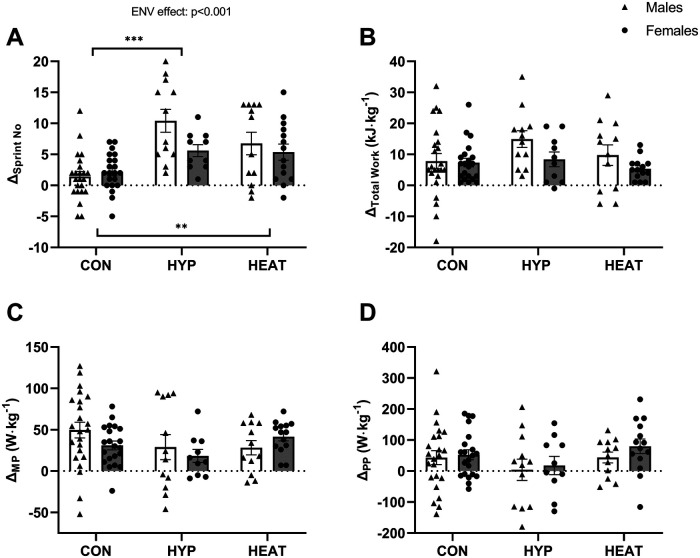
Difference (*Δ*) between the Pre- and post- repeated sprint ability test (RSA) in: **(A)** number of sprints (*Δ*_Sprint No_), **(B)** total work (*Δ*_Total Work_), **(C)** mean power output (*Δ*_MP_) and **(D)** peak power output (*Δ*_PP_), presented by environmental condition, i.e., repeated sprint training in control (CON), hypoxia (HYP), or heat (HEAT), and by sex, i.e., Males and Females. Results are reported to BM. Values are means ± SEM. ***p < 0.001 for difference between CON and HYP, **p < 0.01 for difference between CON and HEAT.

**Figure 3:**
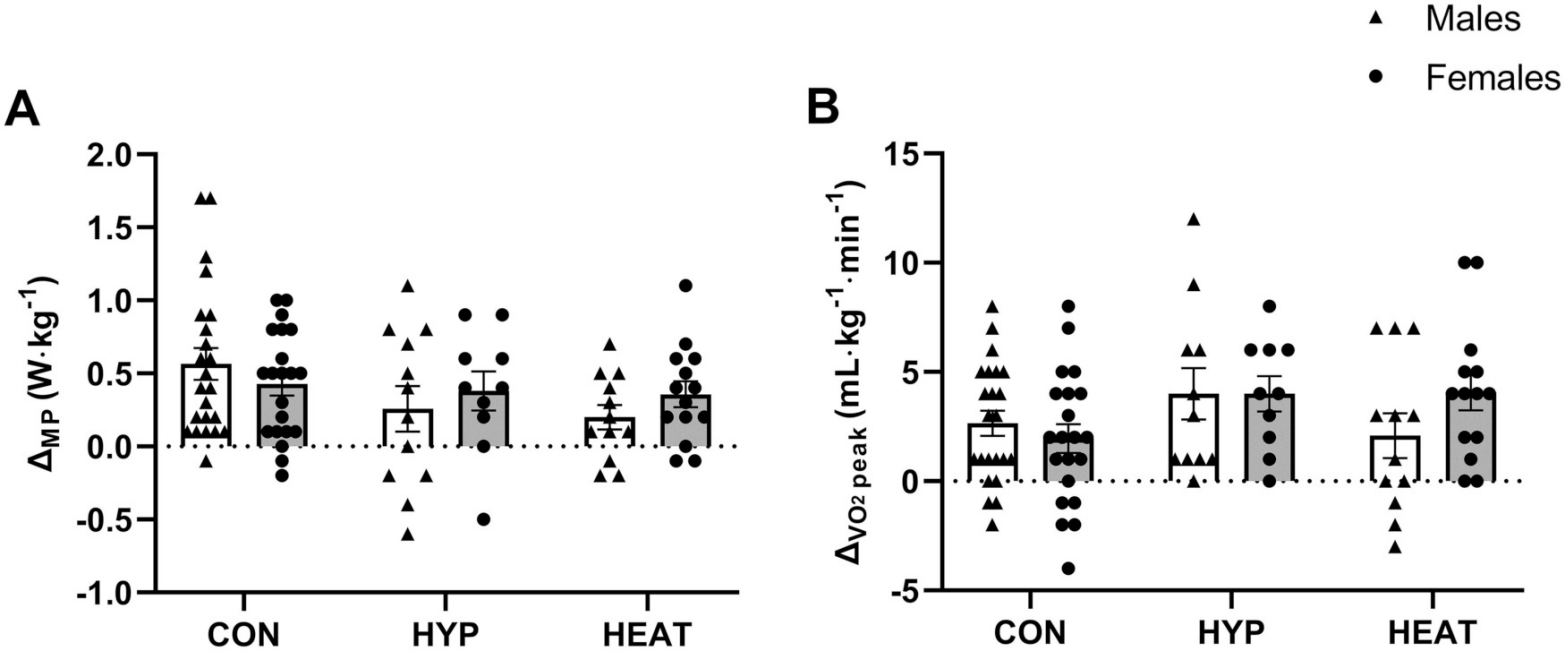
Difference (*Δ*) between the Pre- and post-: **(A)** Wingate test mean power output (*Δ*_MP_) and **(B)** VO_2_peak test (*Δ*_VO2peak_), presented by environmental condition, i.e., repeated sprint training in control (CON), hypoxia (HYP), or heat (HEAT), and by sex, i.e., Males and Females. Results are reported to BM. Values are means ± SEM.

**Figure 4:**
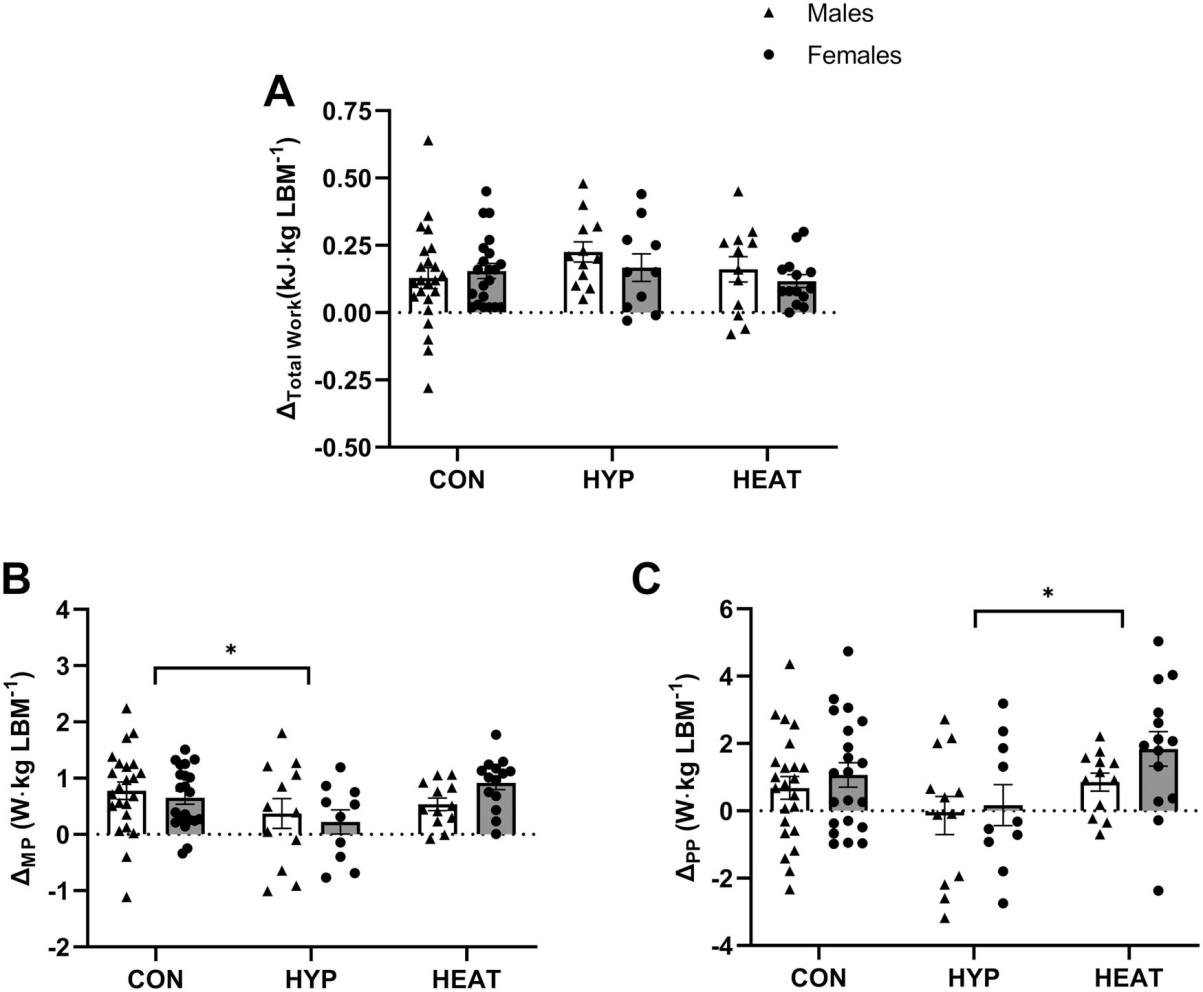
Difference (*Δ*) between the Pre- and post- repeated sprint ability test (RSA) in: **(A)** total work (*Δ*_Total Work_), **(B)** mean power output (*Δ*_MP_) and **(C)** peak power output (*Δ*_PP_), presented by environmental condition, i.e., repeated sprint training in control (CON), hypoxia (HYP), or heat (HEAT), and by sex, i.e., Males and Females. Results are reported to LBM. Values are means ± SEM. ***p < 0.001 for difference between CON and HYP, **p < 0.01 for difference between CON and HEAT.

**Figure 5:**
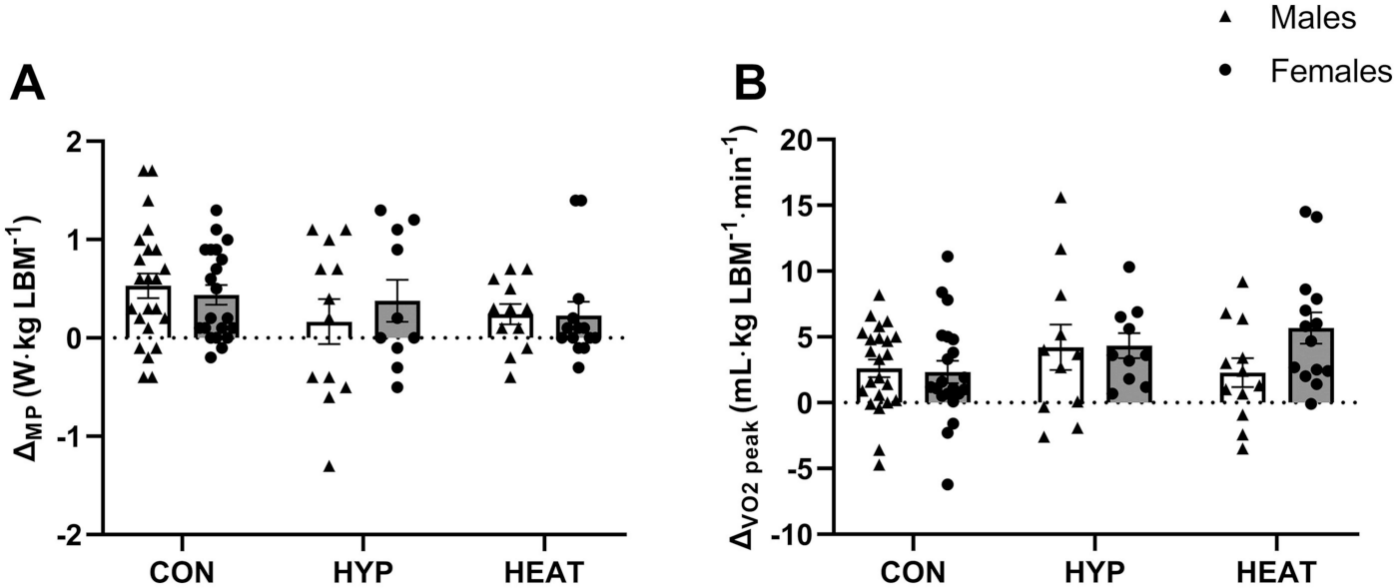
Difference (*Δ*) between the Pre- and post-: **(A)** Wingate test mean power output (*Δ*_MP_) and **(B)** VO_2_peak test (*Δ*_VO2peak_), presented by environmental condition, i.e., repeated sprint training in control (CON), hypoxia (HYP), or heat (HEAT), and by sex, i.e., Males and Females. Results are reported to LBM. Values are means ± SEM.

Except for the number of sprints, the results are reported relative to both BM (Figures 2 and 3) and LBM (Figures 4 and 5).

### 3.1 RSA

The delta in the number of sprints between the Pre- and Post-RSA test (*Δ*_Sprint No_) differed among environmental conditions (main effect of ENV, *p* < 0.001, *η*_p_^2^ = 0.263) (Figure 2A). The *post-hoc* analyses revealed that *Δ*_Sprint No_ was higher in each of the two intervention groups compared to the control group (HYP vs. CON, *Δ*_Sprint No_ = + 6 ± 1 sprints, *p* < 0.001, CI = [−9, −3]; HEAT vs. CON, *Δ*_Sprint No_ = + 4 ± 1 sprints, *p* = 0.001, CI = [−7, −1]). However, there was no difference between the hypoxia and heat group (*p* = 0.371). Moreover, there was a tendency for a main effect of sex (*p* = 0.071, *η*_p_^2^ = 0.012) and for a sex*ENV interaction (*p* = 0.070). *post-hoc* analysis revealed that the *Δ*_Sprint No_ was higher in males in the hypoxic group compared to the other groups (*p* = 0.015; Figure 2A).

The difference in total work, spent during the RSA test, between Pre- and Post- (*Δ* Total Work) did not differ between environmental conditions (relative to BM: *p* = 0.182, *η*_p_^2^ = 0.044, Figure 2B; relative to LBM: *p* = 0.320, *η*_p_^2^ = 0.029, Figure 4A), showing that the improvement in total work did not differ between the control, hypoxic and heat group. In addition, there was a tendency for a main effect of sex when expressed relative to BM (relative to BM: *p* = 0.055, *η*_p_^2^ = 0.031, Figure 2B; relative to LBM: *p* = 0.430, *η*_p_^2^ = 0.003, Figure 4A) but no sex*ENV interaction (relative to BM: *p* = 0.398, Figure 2B; relative to LBM: *p* = 0.457, Figure 4A), indicating that the increase in total work tended to be higher in males than females overall, but did not differ between conditions.

In terms of power output produced during the repeated sprints of the RSA test, there was a main effect of ENV on the *Δ* between Pre- and Post-test in mean or peak power only when expressed relative to LBM (relative to BM: *Δ* MP, *p* = 0.210, *η*_p_^2^ = 0.034, Figure 2C; *Δ* PP, *p* = 0.158, *η*_p_^2^ = 0.044, Figure 2D; relative to LBM: *Δ* MP *p* = 0.032, *η*_p_^2^ = 0.075, *post-hoc* analysis: difference between HYP and CON, *p* = 0.044, Figure 4B; *Δ* PP: *p* = 0.028, *η*_p_^2^ = 0.083, *post-hoc* analysis: difference between HYP and HEAT, *p* = 0.024, Figure 4C), showing that improvements differ between environmental conditions. Moreover, there was no main effect of sex (relative to BM: *Δ* MP, *p* = 0.495, *η*_p_^2^ = 0.011; *Δ* PP, *p* = 0.334, *η*_p_^2^ = 0.012, Figures 2C,D; relative to LBM: *Δ* MP, *p* = 0.800, *η*_p_^2^ = 0; *Δ* PP *p* = 0.136, *η*_p_^2^ = 0.029, Figures 4B,C) or sex*ENV interaction (relative to BM: *Δ* MP, *p* = 0.195, *Δ* PP, *p* = 0.837, Figures 2C,D; relative to LBM: *Δ* MP, *p* = 0.233, *Δ* PP, *p* = 0.727, Figures 4B,C), indicating that increases in power output did not differ between males and females.

### 3.2 Wingate mean power output and $\dot{V}O_{2}$ peak

Similarly to the results of total work and power output of the RSA test, the *Δ* between Pre- and Post-test in Wingate mean power output and $\dot{V}O_{2}$ peak showed no differences between environmental conditions (relative to BM: *Δ* Wingate, *p* = 0.085, *η*_p_^2^ = 0.055, Figure 3A; *Δ* $\dot{V}O_{2}$ peak, *p* = 0.121, *η*_p_^2^ = 0.047, Figure 3B; relative to LBM: *Δ* Wingate, *p* = 0.167, *η*_p_^2^ = 0.043, Figure 5A; *Δ* $\dot{V}O_{2}$ peak: *p* = 0.543, *η*_p_^2^ = 0.018, Figure 5B) or sexes (relative to BM: *Δ* Wingate, *p* = 0.613, *η*_p_^2^ = 0; Figure 3A; *Δ* $\dot{V}O_{2}$ peak, *p* = 0.532, *η*_p_^2^ = 0.001, Figure 3B; relative to LBM: *Δ* Wingate, *p* = 0.795, *η*_p_^2^ = 0, Figure 5A; *Δ* $\dot{V}O_{2}$ peak: *p* = 0.098, *η*_p_^2^ = 0.021, Figure 5B), and no sex*ENV interactions (relative to BM: *Δ* Wingate, *p* = 0.303, Figure 3A; *Δ* $\dot{V}O_{2}$ peak, *p* = 0.221, Figure 3B; relative to LBM: *Δ* Wingate, *p* = 0.584, Figure 5A; *Δ* $\dot{V}O_{2}$ peak, *p* = 0.329, Figure 5B). This indicates that the increases observed in both Wingate mean power and $\dot{V}O_{2}$ peak were consistent across environmental conditions and the two sexes.

## 4 Discussion

To the best of our knowledge, this is the first comparative analysis aiming to determine which of repeated sprint training in hypoxia or in heat is more effective at improving RSA under normoxic and temperate conditions, and whether performance responses and adaptations differ between sexes. Our main finding is that the improvement (delta) in sprint number was similar following RSH and RSHT in males and females. However, it is important to highlight that we observed a tendency for a main effect of sex and a sex*environmental condition interaction. Specifically, *post-hoc* analysis revealed that male participants in the hypoxic group showed the largest delta, e.g., 10 sprints, compared to males in the heat group (7 sprints) and to females (6 in hypoxia and 5 in heat). These results suggest that while both environmental conditions enhance repeated sprint performance, hypoxia may offer a slight advantage, particularly in males. Furthermore, the improvements in aerobic and anaerobic capacities, as well as power output reported to BM during the RSA test demonstrated no differences between environmental conditions, in either males or females, while the improvement in total work tended to be generally higher in males than females. Of note, when reporting power output during the RSA test relative to LBM, lower values were found in HYPOXIA compared to the control group for the mean power and compared to HEAT for the peak power. Those results indicate that environmental conditions may exert differential power regulation during the RSA test when reporting the results to LBM. Globally, when investigating performance and physiological adaptations in men and women, the way results are reported, namely vs. BM or LBM, might reveal critical for the interpretation of the data.

To our knowledge, the only review that has compared the benefits of hypoxic vs. heat training focused on endurance training and performance, which makes direct comparisons between our findings on RSA and that review more difficult. The review by Baranauskas et al. (2021) reported that hypoxic endurance training results in an expansion of red blood cell volume and hemoglobin mass, which directly increases the oxygen transport capacity and therefore improves $\dot{V}O_{2}$ peak and endurance performance in athletes (27). In contrast, the primary adaptation after heat training is the expansion of plasma volume, which enhances endurance performance through an increase in stroke volume and eventually $\dot{V}O_{2}$ peak (28, 29). Non hematological adaptations are also observed with both hypoxic and heat training, including improvements in skeletal muscle buffering capacity and lactate kinetics (29). Nevertheless, an enhanced exercise economy is demonstrated only after training in hypoxia (28). Based on the current evidence in the literature, the authors concluded that while both modalities elicit distinct physiological adaptations, hypoxic training yields more pronounced benefits and is thus considered the preferred environmental stimulus for endurance athletes (12). Our findings align with these conclusions as we showed performance improvements following both training methods, with a tendency for greater RSA enhancements after RSH, particularly among males. It is important to highlight, however, that the performance improvements observed after RSH and RSHT are more likely attributable to muscular adaptations, as detailed in Piperi et al. 2024 (22) and in Piperi et al. (2026) (23), rather than to central adaptations reported in the review by Baranauskas et al. (2021).

Repeated sprint exercise depends on both aerobic and anaerobic capacities (30). In later sprints, oxidative phosphorylation can contribute up to 40% of energy, which may explain the observed global improvements in $\dot{V}O_{2}$ peak in both the HYPOXIA and the HEAT study (31). However, the delta in Pre- to Post- $\dot{V}O_{2}$ peak did not differ between conditions, indicating that the addition of hypoxia or heat provided no further benefits in aerobic capacity in either sex. Similarly, the improvement in Wingate mean power output was comparable between control, hypoxic and heat conditions in males and females. Our findings are in line with Faiss et al. (2013), who demonstrated that both RSN and RSH improved Wingate performance to the same extend in males (9). Conversely, a recent study by Maciejczyk et al. (2024) reported that hypoxia further improved peak but not mean Wingate power output compared to training in normoxia in males (32). Heat, however, did not affect anaerobic power. Interestingly, this study also examined the effects of concurrent hypoxic and heat training on anaerobic capacity. Results suggest that heat added to hypoxia did not further increase power output. We should note that this study used an interval training design, which consists of longer efforts and recovery periods, making it slightly different to our repeated sprint training protocol. Other studies that have used a repeated sprint training protocol similar to ours have also investigated the effect of combined environmental stressors on RSA (33). They reported that although hypoxia and heat separately improved power output during an RSA test, adding heat stress to RSH did not further enhance performance. Yamaguchi et al. (2023) even reported that the addition of heat to RSH may mask muscle tissue adaptations by attenuating the hypoxic stimulus (33). More specifically, hypoxia in RSH alone lowers oxygen saturation (SpO_2_) and subsequently muscle partial pressure (PO_2_), which is a crucial factor for triggering adaptations, including pH regulation, enhanced blood perfusion and increased oxygen extraction (9). Adding thermal stress may increase ventilation and reduce blood partial pressure of carbon dioxide, potentially limiting the rightward shift in the oxygen-hemoglobin dissociation curve (34). Therefore, training under combined hypoxic and hot conditions is not recommended for enhancing RSA.

We should highlight that most of the aforementioned research on RSH and RSHT has been conducted in males, with no studies to date directly comparing the effects of those training methods between sexes. Interestingly, men and women in our studies showed comparable performance improvements and adaptations following RSH and RSHT. However, several physiological sex differences have been reported in our studies and in the literature (35–37), suggesting that the underlying mechanisms behind similar performance improvements between sexes may not be entirely the same. For example, our work showed that absolute power output during the exercise performance tests RSA, Wingate and $\dot{V}O_{2}$ peak and relative $\dot{V}O_{2}$ peak were overall higher in men than women. Consequently, total work, which depends on sprint number and power output, tended to be higher in men than women. Women generally have lower absolute and relative muscle mass than men, and higher fat percentage, which contributes to lower power output during cycling exercise (37). Also, women have greater distribution and relative area of type I fibers than men (36). These fibers produce less force and rely more on aerobic metabolism because of greater capillary density, higher mitochondrial volume, and increased oxidative enzyme activity (38). As a result, women have a higher fatigue threshold and greater endurance than men, when sprints are repeated (39). The lower relative $\dot{V}O_{2}$ peak reported in females could be explained by differences in the cardiovascular and respiratory systems, such as smaller heart size, lower hemoglobin levels, reduced blood volume (40, 41), smaller lungs, narrower airways, and lower maximal ventilation (42). Sex differences in neuromuscular activation play also play a role in the greater fatigue resistance observed in females (43). Since RSA depends also on neural factors (30), these differences may influence performance outcomes across sexes in distinct ways.

Of note, the number of sprints during the RSA test in the HYPOXIA study tended to increase more from Pre- to Post-test in men than women, suggesting that men had higher capacity for improvements than women, after RSH. This could be related to sex differences in exercise-induced hypoxemia. Indeed, some evidence support that females may experience greater exercise-induced hypoxemia than males when exposed to hypoxic conditions, most likely due to their lower hemoglobin concentrations and smaller lung volumes (14). However, we observed no sex differences in RSA improvements following RSHT, despite the higher sweat rate reported in men at both Pre- and Post-test. A main difference between sexes regarding their response to heat acclimation lies within the time course of adaptations, with females requiring longer protocols than males to achieve similar reductions in thermoregulatory strain (18). Our 7-week protocol, however, seemed to be long enough to provoke similar adaptations in females compared to males.

## 5 Limitations and conclusions

Despite the rigor of the experimental protocol and analyses, our work presents certain limitations that are worth acknowledging. To begin with, the number of female participants in the hypoxic group of the HYPOXIA study was not optimal, since the predicted number required to detect an effect was 12 per group instead of 10. Although this sample size was adequate to show a significant improvement in RSA in females, the desired number of females was not fully reached due to the larger difficulties to recruit them compared to males.

Additionally, the resistance of the RSA and Wingate tests was determined based on body mass as in previous literature (9, 44), and not LBM, which resulted in a significantly higher relative torque in females compared to males when expressed in Nm·kg^−1^ of LBM. This discrepancy contributed, in part, to the large sex difference in the number of sprints completed during the RSA test at baseline and Post-test in both studies, and it may have also influenced the Wingate test results. Although males and females seem to be well matched in the HYPOXIA study in terms of weekly training volume and physical fitness, it was not the case in the HEAT study. In the latter, males in the heat group had higher baseline $\dot{V}O_{2}$ peak values, even when expressed relatively to LBM, and higher training volume than females in the control group, which may be another factor explaining the sex-differences in RSA performance at baseline and at Post-test. This limitation highlights the importance of using LBM-adjusted testing protocols to allow more accurate comparisons between sexes. In the same line, small differences were found when reporting the results from the RSA test relative to LBM compared to BM. It is thus important to consider which of BM or LBM needs to be used to adjust the load during the tests but also to report the results.

Moreover, the sample of females in our studies was heterogenous, as it included females both with and without hormonal contraception. The hormonal profile differs between hormonal contraception users and non-users, which may impact exercise performance (45). Moreover, due to logistic reasons, it was not feasible to test all female participants during their early follicular phase of the menstrual cycle when sex hormones are low and stable (46). However, the analyses of 17β-estradiol levels show that they did not differ between Pre- and Post-test in both the HYPOXIA and HEAT studies. Also, growing evidence suggests that menstrual cycle does not significantly impact athletic performance (46).

To conclude, this comparative analysis demonstrates that seven weeks of repeated sprint training performed twice per week in either hypoxia (3,000 m) or heat (30 °C and 60% RH) can equally improve RSA under normoxic and temperate conditions in both males and females, with a tendency for greater performance improvements in males following hypoxic training.

## 6 Perspectives

From a practical perspective, we propose that female recreational athletes can use either RSH or RSHT to improve RSA in normoxic and temperate conditions since the two protocols provide equal benefits to this population. However, our work did not examine the molecular or metabolic mechanisms responsible for the performance improvements we observed. While those mechanisms were investigated in males following RSH (8, 9), information in females is lacking. Moreover, the molecular and metabolic adaptations following RSHT in both males and females have received limited attention. Future studies are needed in this research area to help us better understand the mechanisms behind the performance improvements following repeated sprint training in hypoxia or heat.

## Data Availability

The original contributions presented in the study are included in the article/Supplementary Material, further inquiries can be directed to the corresponding author/s.
